# Predicting antibiotic prescription after symptomatic treatment for urinary tract infection: development of a model using data from an RCT in general practice

**DOI:** 10.3399/bjgp16X684361

**Published:** 2016-03-11

**Authors:** Ildikó Gágyor, Jörg Haasenritter, Jutta Bleidorn, Warren McIsaac, Guido Schmiemann, Eva Hummers-Pradier, Wolfgang Himmel

**Affiliations:** Department of General Practice, Göttingen University Medical Center, Göttingen, Germany.; Department of General Practice/Family Medicine, Philipps University Marburg, Marburg, Germany.; Institute of General Practice, Hannover Medical School, Hannover, Germany.; Ray D Wolfe Department of Family Medicine, Mount Sinai Hospital, Toronto, Ontario, Canada.; Institute for Public Health and Nursing Research, Department for Health Services Research, University of Bremen, Bremen, Germany.; Department of General Practice, Göttingen University Medical Center, Göttingen, Germany.; Department of General Practice, Göttingen University Medical Center, Göttingen, Germany.

**Keywords:** family practice, ibuprofen, prediction model, urinary anti-infective agents, urinary tract infections

## Abstract

**Background:**

Uncomplicated urinary tract infection (UTI) is often treated with antibiotics, resulting in increasing resistance levels. A randomised controlled trial showed that two-thirds of females with UTI treated symptomatically recovered without subsequent antibiotic treatment.

**Aim:**

To investigate whether there are differences between females with a UTI who were subsequently prescribed antibiotics and those who recovered with symptomatic treatment only, and to develop a model to predict those who can safely and effectively be treated symptomatically.

**Design and setting:**

This is a subgroup analysis of females assigned to ibuprofen in a UTI trial in general practices.

**Method:**

Multiple logistic regression analysis was used to select variables for a prediction model, The discriminative value of the model was estimated by the area under the receiver operator curve (AUC) and the effects of different thresholds were calculated within the model predicting antibiotic prescription and need for follow-up visits.

**Results:**

Of the 235 females in the ibuprofen group, 79 were subsequently prescribed antibiotics within 28 days of follow-up. The final model included five predictors: urgency/frequency, impaired daily activities, and positive dipstick test results for erythrocytes, leucocytes, and nitrite. The AUC was 0.73 (95% CI = 0.67 to 0.80). A reasonable threshold for antibiotic initiation would result in 58% of females presenting with UTI being treated with antibiotics. Of the remaining females, only 6% would return to the practice because of symptomatic treatment failure.

**Conclusion:**

The present model revealed moderately good accuracy and could be the basis for a decision aid for GPs and females to find the treatment option that fits best.

## INTRODUCTION

Urinary tract infections (UTI) in females are common and are usually treated with antibiotics.^1^,^2^ Consequently, the number of antibiotic prescriptions for UTI in general practice is high and is linked with increasing antimicrobial resistance, resulting in a call for action.^3^ Lowering antibiotic prescription rates in general practice is a promising approach for reduction of antimicrobial resistance.^4^–^6^

Different strategies have proven successful to reduce antibiotic prescriptions for respiratory tract infections.^4^,^7^,^8^ As for uncomplicated UTI, both the effectiveness of symptomatic treatment in females with uncomplicated UTI^9^ and delayed antibiotic treatment have been tested in randomised controlled trials.^10^ It was shown recently that symptomatic treatment can substantially reduce the number of antibiotic courses.^9^ It would be helpful to be able to predict which females have a high risk of therapeutic failure if treated symptomatically only, and to identify those who can safely and effectively be treated with symptomatic treatment alone.

It was hypothesised that females with UTI who recover without antibiotics have different characteristics and symptom experience from females who need antibiotic treatment. The aim of this analysis was to identify such determinants and to develop a prediction model to find the best treatment option for females with a UTI.

## METHOD

### Context and study design

Data analysis and the development of the prediction model are based on data from the immediate versus conditional antibiotic treatment for women with UTI (ICUTI) trial.^11^ This randomised trial assessed the comparative effectiveness of a symptomatic treatment approach (ibuprofen for 3 days) and antibiotics if needed, with immediate antibiotic treatment with fosfomycin in females with uncomplicated UTI.^9^,^11^ All females were instructed to consult their GP again in case of persistent or worsening UTI symptoms or any UTI-related complications (that is, pyelonephritis). For these patients, antibiotic treatment was prescribed at the discretion of GPs.

Only data for females assigned to the ibuprofen group in the ICUTI trial were used. Of these, only one-third subsequently required antibiotics because of persistent or recurrent symptoms in the following 28 days, with the other two-thirds recovering without any antibiotics. Compared with females treated with antibiotics immediately, the group investigated here had a higher symptom burden; however, the biggest difference of daily severity of symptoms was 1 point on a 12-point symptom sum score (3.20 ± 2.50 versus 2.20 ± 2.00) at day 2. Females in the ibuprofen group had a longer duration of symptoms of 1 day. Pyelonephritis occurred more frequently in the ibuprofen group (5 versus 1, *P* = 0.122). Of females treated subsequently with antibiotics, 38% consulted their GPs within the first 3 days and 38% on days 4–7 after inclusion.

**How this fits in**

A randomised controlled trial showed that many females with UTI recover with symptomatic treatment only, but approximately one-third required antibiotics because of symptomatic treatment failure. A rule of thumb and appropriate information on effective and safe treatment options for UTI would help both GPs and patients. Considering UTI-related symptoms, dipstick test results for erythrocytes, leucocytes, and nitrites, and impairment of activity, a model was developed and validated to predict which females would most likely benefit from antibiotic treatment.

For the current analysis, the ibuprofen group was split into two subgroups: patients who recovered without any antibiotics (the No Antibiotic group) and patients who received an antibiotic for UTI within the following 28 days (the Antibiotic group).

### Participants

Females aged 18–65 years with dysuria, urgency and frequency of micturition, or lower abdominal pain were enrolled in the ICUTI study. Key exclusion criteria were UTI within the previous 2 weeks, temperature >38°C, upper UTI, pregnancy, renal diseases and urine catheterisation, history of gastrointestinal ulcers and severe conditions, and treatment with non-steroidal anti-inflammatory drugs (NSAIDs) or antibiotics.^11^

### Development of the prediction model

The outcome to be predicted was whether or not a female treated symptomatically subsequently received an antibiotic within 4 weeks of initial presentation at the practice. As potential predictors, variables were considered that were available at initial presentation to the practice, that is, age, number of previous UTIs, symptom duration at inclusion, UTI symptoms (dysuria, urgency or frequency of micturition, and low abdominal pain), activity impairment, and results of the dipstick test (nitrite, leucocytes, erythrocytes). For the analysis, urgency or frequency were defined as one symptom in accordance with the German general linguistic usage (*‘häufiger Harndrang’*, that is, frequent urinary urgency). Urine culture test was not included in the multivariate analysis because results are not available at presentation.

Participating females scored UTI symptoms on a 5-point scale from 0 (not at all) to 4 (very strong/frequent). A female’s impairment was measured by the Activity Impairment Assessment (AIA), a 5-item score (range 0–4) according to the time during which a patient’s work or personal activities have been impaired because of UTI.^12^ Scores were dichotomised for each symptom in mild symptoms (0–1 point) and moderate-to-strong symptoms (2–4 points). The single items of AIA are rather similar and can be summed to one score (range 0–20). Scores >10 were considered to be impairment at least most of the time. There were no patients with extremely differing scores for one or two items of the AIA compared with the remaining four or three items.

### Statistical analysis

Absolute and relative frequencies of each potential predictor were determined in both subgroups and odds ratios (ORs), and their corresponding 95% confidence intervals (CI) were calculated. The predictors were checked for multicollinearity, that is, a high correlation between two or more variables.

A multivariate logistic regression analysis was performed to select significant variables for the final prediction model. All clinically important variables were included in the multivariate analysis irrespective of the results of the univariate analysis. The least absolute shrinkage and selection operator (LASSO) approach was used, accounting for overfitting by shrinking the regression coefficients towards 0.^13^ The optimal penalty value λ was identified using leave-one-out-cross validation.^14^ A linear point score was constructed by multiplying the regression coefficients for each predictor variable by 100 and summing the total.

To assess the accuracy and the discriminative value of the final prediction model, sensitivity was plotted against the false-positive rate (1 – specificity) over a range of cut-point values for the continuous linear score in the receiver operating characteristic (ROC) space, and the area under the ROC curve (AUC) was calculated.^13^ For several cut-point values, 2 by 2 tables were created, and the sensitivity and specificity and the respective CIs were calculated using the Wilson score method.^15^ To evaluate clinical utility, for each of these cut-point values the proportions of patients were calculated who would receive antibiotics at presentation according to the model, and who would be initially classified as not requiring antibiotic treatment but subsequently return to the practice because of symptomatic treatment failure (that is, persistent, worsening, or recurrent symptoms). Bootstrap resampling was used to assess the over-optimism of the final model,^13^ and the optimism-corrected AUC of the final model was determined.

The glmnet pROC, and boot packages of the R 3.0.3 programme.

## RESULTS

**Figure 1.**
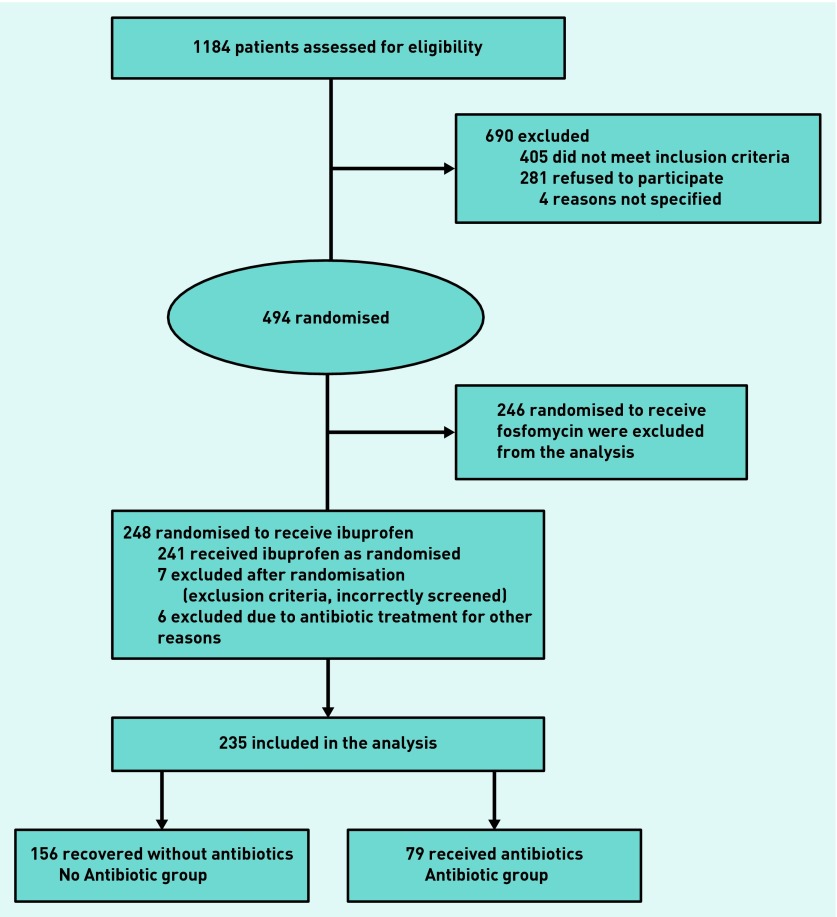
***Participant flow.***

Of the 235 females included in the current analysis, 79 (34%) were prescribed an antibiotic prescription for UTI-related symptoms within 28 days of initial consultation, and 156 (66%) were not (Figure 1). Females in the Antibiotic group were somewhat older (39 versus 37 years) and nearly all of them (94%) had a positive urine culture compared with 66% in the No Antibiotic group, resulting in a rather high OR of 7.7 (95% CI = 2.93 to 20.21; Table 1). Five females in the Antibiotic group were diagnosed with pyelonephritis.

**Table 1.** Predictors for a subsequent antibiotic prescription

**Variable**	**Antibiotics**	**No antibiotics**	**Unadjusted**	
		
	***N* = 79, %**	***N* = 156, %**	**OR**	**95% CI**
Age, years, mean (SD)	38.9 (15.2)	36.6 (14.4)	1.0	0.99 to 1.03
Symptom duration at inclusion, >2 days	35.4	35.3	1.0	0.57 to 1.78
Recurrent UTI^a^	15.2	18.6	0.8	0.38 to 1.64
Dysuria (at least moderate)	84.8	77.6	1.6	0.79 to 3.32
Urgency/frequency (at least moderate)	84.8	71.2	2.3	1.12 to 4.58
Low abdominal pain (at least moderate)	40.5	43.6	0.9	0.51 to 1.53
Regular daily activities impaired (at least most of the time)	55.7	43.0	1.7	0.97 to 2.88
Nitrite positive	31.7	16.0	2.4	1.28 to 4.59
Leucocytes positive	94.9	79.5	4.8	1.65 to 14.22
Erythrocytes positive	89.9	66.0	4.6	2.05 to 10.19
Urine culture positive^b^	93.7	65.8	7.7	2.93 to 20.21

a Recurrent UTI refers to preceding UTI within the previous year.

b N = 152, four urine culture results were missed.

SD = standard deviation.

Of all reported UTI-related symptoms, moderate-to-severe urgency/frequency was the strongest predictor in the univariate analysis for subsequent antibiotic treatment (OR 2.3, 95% CI = 1.12 to 4.58). Other significant predictors were positive dipstick test results for erythrocytes, leucocytes, and nitrite (Table 1). Impairment of activity ‘most of the time’, dysuria, and low abdominal pain did not show significant association with subsequent antibiotic treatment. The same was true for age, symptom duration at inclusion, and recurrent UTI. The two-sided correlation of all predictors was <0.6, indicating no substantial multicollinearity.

The final model included five predictors: moderate-to-severe urgency or frequency, positive dipstick test results for erythrocytes, leucocytes, and nitrite, and impairment of daily activities (Table 2). The sum of the scores ranged from 0–294 points as shown in Table 2.

**Table 2.** Regression coefficients and points assigned to each predictor

**Predictor**	**Regression coefficients**	**Points**
(Intercept)	−2.682	NA
Age, mean (SD)	0.00	–
Symptom duration at inclusion, >2 days	0.00	–
Recurrent UTI^a^	0.00	–
Dysuria (at least moderate)	0.00	–
Urgency/frequency (at least moderate)^b^	0.50	50
Low abdominal pain (at least moderate)	0.00	–
Regular daily activities impaired (at least most of the time)^b^	0.19	19
Nitrite positive^b^	0.56	56
Leucocytes positive^b^	0.75	75
Erythrocytes positive^b^	0.94	94

a Recurrent UTI refers to preceding UTI within the previous year.

b Predictors included in the final model.

SD = standard deviation.

**Figure 2.**
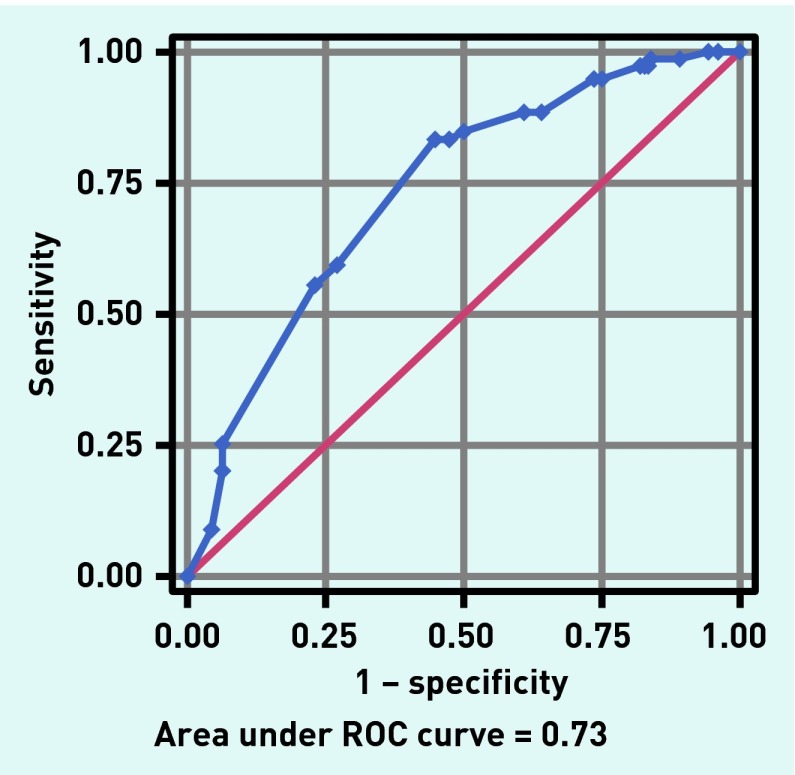
***Receiving operator curve (ROC) for the final model.***

Figure 2 shows the ROC with an AUC of the final model of 0.73 (95% CI = 0.67 to 0.80). The optimism-corrected AUC was 0.69.

The accuracy of prediction for a couple of cut-off points was calculated (Table 3). For example, if a score of ≥210 points is required for antibiotic treatment, 58% of all females presenting with UTI would be advised to be treated with antibiotics. Vice versa, in 42% of the females the GP would not recommend antibiotics. This score has a rather high sensitivity of 84%, implying that only 6% of the females started on symptomatic treatment would return to the practice because of treatment failure (Table 3). Specificity is 55%, however, implying that a rather large group of patients who could probably manage their condition without antibiotics is not recognised. If the cut-off is <210, that is only few predictors must be ‘positive’, specificity will also be low. In this case, more females would be advised initially to take antibiotics and fewer females are expected to subsequently return to the practice.

**Table 3.** Measures of classification accuracy for several cut-off values to predict an antibiotic need

**Cut-off point value**	**Sensitivity, % (95% CI)**	**Specificity, % (95% CI)**	**Antibiotics^a^ prescribed, % (95% CI)**	**Returning^b^ patients, % (95% CI)**
≥60	100.0 (95.4 to 100.0)	5.8 (3.1 to 10.6)	96.2 (92.9 to 100.0)	0.0 (0.0 to 1.6)
≥120	97.5 (91.2 to 99.3)	17.9 (12.7 to 24.7)	87.2 (82.4 to 91.0)	0.9 (0.02 to 3.0)
≥165	88.6 (79.8 to 93.9)	39.1 (31.8 to 46.9)	70.2 (64.1 to 75.7)	3.8 (2.0 to 7.1)
≥210	83.5 (73.9 to 90.1)	55.1 (47.3 to 62.7)	57.9 (51.5 to 64.0)	5.5 (3.3 to 9.2)
≥220	59.5 (48.5 to 69.6)	73.1 (65.6 to 79.4)	37.9 (31.9 to 44.2)	13.6 (9.8 to 18.6)
≥230	55.7 (44.7 to 66.1)	76.9 (69.7 to 82.8)	34.0 (28.3 to 40.3)	14.9 (10.9 to 20.0)
≥240	25.3 (17.0 to 35.9)	93.6 (88.6 to 96.5)	12.8 (9.1 to 17.6)	25.1 (20.0 to 31.0)

Cases are classified by the model as ‘negative’ if point score < threshold; cases are classified by the model as ‘positive’ if point score ≥ threshold.

a Antibiotics prescribed refers to the number of positive cases (= patients that the model predicted will receive an antibiotic) in relation to all patients.

b Returning patients refers to the number of false-negative cases (= patients that the model falsely predicted will not receive an antibiotic) in relation to all patients.

In the present dataset, five females returned to the practice with an upper UTI (pyelonephritis). At their initial presentation, two had yielded a score of 219 and three had presented with scores of ≥238 in the model.

## DISCUSSION

### Summary

The present prediction model for subsequent antibiotic prescriptions after initial symptomatic treatment included five factors: a positive test result for nitrite, leucocytes, and erythrocytes, moderate-to-severe urgency or frequency, and impairment of regular daily activities most of the time. A threshold score of 210 for initial antibiotic treatment yielded a high accuracy in prediction, with a significant reduction of antibiotic prescriptions in females presenting with UTI and a relatively small number of females who would return to the practice because of symptomatic treatment failure. It should be considered, however, that the effect size of these five different factors was rather low compared with the predictive power of urine culture tests. This is also evident in an optimism-corrected AUC of only 0.69 for the final predictive model.

### Strengths and limitations

To the best of the authors’ knowledge, this is the first follow-up study of females who initially received symptomatic treatment with ibuprofen for a UTI, and the first prediction model to predict those who can safely and effectively be treated with symptomatic treatment alone. This model may encourage patients and GPs to first treat UTI symptomatically.

The study sample was a subgroup of a randomised controlled double-blinded trial including females with an upper age limit and further inclusion and exclusion criteria. Participating females had to agree to be randomised to symptomatic treatment, which may have resulted in some selection bias. Baseline data (that is, proportion of verified UTI by urine cultures) were comparable, however, with data from previous studies.^10^,^16^,^17^

The Antibiotic group in this analysis comprised females who received subsequent antibiotic treatment after initial symptomatic treatment with ibuprofen during the ICUTI trial. There is no detailed information available about the reasons for the decision to subsequently prescribe antibiotics, or on whether the decision was discussed between patient and GP. Both patients and GPs may have been influenced by the study design. Uncertainty concerning the trial drug because of blinding and continuous monitoring of symptoms may have enhanced the re-consultation rate and, consecutively, the number of antibiotic prescriptions. GPs who made the final decision to prescribe antibiotics were not blinded to the predictors used in the model, but had no knowledge of the prediction model itself. If females enrolled in the ICUTI trial visited the practice again for UTI reasons after a few days, the treatment choice was supported by the result of the urine culture. Transferability of the results into a prediction model may therefore be limited. Knowing culture results could have influenced decisions from both GPs and patients, resulting in an overestimation of the predictive performance of the model. However, only four females received antibiotics despite initially negative urine culture results so the probability of a considerable overestimation is low.

The threshold of the present model was set to the number of females who would return to the practice with persistent or recurrent symptoms being low. However, trials have shown (ICUTI) that a considerable proportion of the control group (14%) who were treated with antibiotics at presentation received a further antibiotic prescription.^9^ This shows that even an antibiotic-first approach has a considerable risk of failure.

Data on other UTI-associated factors such as absence of vaginal discharge, smelly and cloudy urine, or back pain were not collected in the ICUTI trial, and were not evaluated in the present analysis.

### Comparison with existing literature

Previous research aiming to reduce unnecessary antibiotic prescriptions focused on diagnostic accuracy of UTI.^17^–^21^ Results of these analyses are contradictory: in some of them, dipstick test results alone were shown as having a moderate value to predict UTI compared with clinical signs.^19^,^20^ Little and colleagues developed a dipstick decision rule and a clinical decision rule separately. Both showed moderate sensitivity and specificity to predict UTI.^21^ In other analyses, urine test results were seen as more relevant than any other clinical data.^17^,^18^ Furthermore, a systematic review showed that the probability of UTI increases if symptoms and signs are combined with dipstick test results.^17^ The present findings are in line with these results: dipstick test results had a greater weighting impact on the regression analysis than did symptom severity and impaired activity. From a clinical perspective it seemed reasonable to include both in the final model.

GPs tend to overestimate the need for antibiotics, and to prescribe them rather than to forgo prescribing.^22^ The concept of delayed prescription proved to be effective in general practice, however, showing the willingness of GPs and patients to lower the number of antibiotic prescriptions.^10^ Furthermore, in many European countries, point-of-care tests for urine culture with results available 24 hours after presentation are popular in general practice.^23^ These approaches may contribute to the reduction of antibiotic prescribing, especially when combined with symptomatic treatment and a decision aid.

### Implications for research and practice

Further research with an independent patient sample is needed to prove whether the present prediction model, although exhibiting a reasonable accuracy, may be adequate for general practice and, if so, which cut-off value may satisfy the needs of females with UTI symptoms and contribute to a more rational handling of antibiotics. A cut-off value of 210 points may be reasonable, with only 58% of females being treated initially with an antibiotic and 6% of the females without an initial antibiotic prescription having to return to the practice. Two out of five females with pyelonephritis had scores of 219, however, which were above the selected threshold of 210 points.

GPs and patients may appreciate the prediction model to discuss different treatment approaches. For example, if both favour symptomatic treatment, they could arrange a wait-and-see approach, combined with appropriate information for the patient or with a delayed prescription of antibiotics. Appropriate information should include the probability of a somewhat higher symptom burden. If culture of urine samples is necessary, symptomatic treatment could bridge the time until results are available^9^ and seek to combat pain, the most important symptom of UTI.^24^

In conclusion, this prediction model may be helpful for GPs and patients to identify those who are likely to recover effectively and safely with symptomatic treatment alone. It can be used especially when females favour non-antibiotic treatment or accept a delayed prescription.^10^,^25^,^26^ The predictive power of the final model can be considered as fair, but it should be validated in a new and independent cohort of females with UTI symptoms. Furthermore, the model should be tested for its effect on antibiotic prescription rate for UTI as well as overall visit rates and cases of pyelonephritis in practice.
